# Characteristics of individuals who received post‐exposure prophylaxis and HIV seroconversion in Malawi: an analysis of national routine HIV testing data

**DOI:** 10.1002/jia2.26473

**Published:** 2025-06-26

**Authors:** Hannock Tweya, Tiwonge Chimpandule, William Wu, Leah Goeke, Zhouyun Zheng, Stone Mbiriyawanda, Tobias Masina, Washington Ozitiosauka, Martha Muyaso, Anna Drabko, Dominik Bilicki, Jiehua Chen, Rose Nyirenda, Andreas Jahn

**Affiliations:** ^1^ International Training and Education Center for Health (I‐TECH) Lilongwe Malawi; ^2^ Department of Global Health University of Washington Seattle Washington USA; ^3^ Directorate of HIV/AIDS, STI and Viral Hepatitis (DHA) Ministry of Health Lilongwe Malawi; ^4^ Quantitative Engineering Design (QED.ai) Lilongwe Malawi

**Keywords:** post‐exposure prophylaxis, HIV seroconversion, HIV testing service, young adult, HIV infection, HIV risk

## Abstract

**Introduction:**

In Malawi, where HIV prevalence remains high at 6.7%, post‐exposure prophylaxis (PEP) has been implemented as one of the HIV prevention strategies. However, there is limited data on the characteristics of PEP users and HIV seroconversion. Using national routine HIV testing services (HTS) programme data, we described the demographic characteristics and risk of exposure to HIV for HTS clients reporting PEP use and determined HIV seroconversion rates among those with baseline HIV‐negative results.

**Methods:**

We conducted a descriptive cross‐sectional study of individuals aged 2 years and older accessing HTS who reported PEP use. A subset was included in a retrospective cohort to determine HIV seroconversion rates. The risk of exposure to HIV was classified as high, ongoing, low and not assessed. HTS encounters data were extracted from a national HTS data repository. Some HTS clients had multiple HTS encounters. Descriptive statistics were reported for the study populations and Poisson regression model with an offset was used to estimate HIV seroconversion rates.

**Results:**

Between November 2022 and July 2023, there were 21,298 HTS encounters where PEP use was reported any time prior. Of the 21,298 encounters, 1847 (8.7%) HTS clients with a baseline HIV‐negative status were included in the cohort study component. The median follow‐up time was 30 days (interquartile range 30–61). Of the 1847 HTS clients, 1055 (57.1%) were males and 928 (50.2%) were aged 20 and 29 years. A total of 329 (17.8%) HTS clients reported a high‐risk HIV exposure event in the past 3 months, 581 (31.5%) had an ongoing risk of exposure to HIV, 892 (48.3%) had low risk of exposure to HIV and 45 (2.4%) assessment was not done. Overall, five individuals seroconverted, yielding a seroconversion rate of 2.08 (0.87−4.99) per 100 person‐years.

**Conclusions:**

The majority of PEP users were young adults and males. A sizeable proportion had an ongoing risk of exposure to HIV. The HIV seroconversion rate was high. Targeted efforts should focus on promoting condom use, encouraging partner testing and expanding access to PEP for those with ongoing HIV exposure.

## 1 INTRODUCTION

HIV acquisition remains a significant public health concern in sub‐Saharan Africa (SSA). Approximately 25.9 million people were living with HIV (PLHIV) in SSA in 2023, which accounted for 67% of the world's PLHIV [1]. Malawi, one of the SSA countries, has one of the highest numbers of PLHIV. As of 2023, the national adult HIV prevalence was estimated at 6.7%, with around 1 million PLHIV. While Malawi has made significant progress towards the UNAIDS 95‐95‐95 targets—95% of PLHIV knew their status, 91% of those diagnosed received sustained antiretroviral therapy (ART) and 87% of those on ART achieved viral suppression—Malawi reported over 12,000 new PLHIV in 2023 [1]. Reducing HIV acquisitions requires a multi‐faceted approach, integrating prevention strategies such as post‐exposure prophylaxis (PEP), pre‐exposure prophylaxis (PrEP), expanding access to voluntary medical male circumcision and promoting consistent condom use [2].

PEP is recommended by the World Health Organization (WHO) to prevent new acquisitions, particularly for individuals at high risk of HIV transmission after exposure [3]. PEP is a short‐term antiretroviral treatment that reduces the risk of HIV acquisition after exposure to HIV‐infected blood. PEP has an estimated 80% effectiveness in preventing HIV acquisition if initiated within 72 hours of exposure, and the entire 28‐day course is completed [3]. However, delayed initiation, incomplete adherence, and repeated high‐risk exposures have raised concerns about its effectiveness, particularly in resource‐limited settings like Malawi [4].

In 2016, the Malawi HIV programme aligned its PEP regimen with the standard first‐line ART regimen for adults and children [5]. The 2022 national HIV guidelines for PEP service include a baseline HIV test—contingent on the availability of testing services—to confirm negative status, provision of a 30‐day supply of PEP and adherence assessment at 30 days. Follow‐up HIV tests are recommended at 3 and 6 months. Data collection on PEP is limited to initiation, with no dedicated tools for follow‐up after the completion of the regimen, leading to a lack of data on HIV seroconversion [6].

In 2021, the Malawi HIV programme introduced a scannable integrated HIV testing register using ScanForm, an artifical intelligence (AI)‐powered technology to digitize and analyse handwritten data, developed by Quantitative Engineering Design (QED.ai) [7]. The integrated HIV testing register also serves as a proxy PEP follow‐up tool to capture information about previous HIV test results, time since the last HIV test, previous PEP use and time since the last PEP use. Despite the implementation of the integrated HIV testing register, the data has not yet been evaluated to understand the characteristics of PEP users and assess HIV seroconversion rates among individuals who access HIV testing services (HTS). Therefore, this study analysed the data to (1) describe the demographic characteristics and risk of exposure to HIV among HTS clients who reported PEP use; and (2) determine HIV seroconversion rates among HTS clients with a baseline HIV‐negative test result who reported previous PEP use.

## 2 METHODS

### 2.1 Study design

We conducted a descriptive cross‐sectional study to profile HTS individuals aged 2 years and older who reported PEP use in Malawi, with a subset of these HTS clients included in a retrospective cohort to determine HIV seroconversion rates. We used national HTS programme data collected between November 2022 and July 2024.

### 2.2 Setting

#### 2.2.1 PEP services and HTS

PEP services are offered in health facilities, some of which have one‐stop centres that provide comprehensive services comprising medical treatment, social welfare, law enforcement and counselling to victims of sexual or gender‐based violence [8]. Eligibility for PEP is determined based on the risk of exposure to HIV. Adults and children (ages < 12 years and below) who experience potential risk of exposure to HIV through occupational (e.g. needlestick injuries in healthcare settings) or non‐occupational events (unprotected consensual sexual intercourse, sexual assault or sharing needles) are considered for PEP. The assessment includes evaluating the timing of the exposure, with PEP being most effective when initiated within 72 hours. Children receive distinct legal and social services from adults.

Clients who are eligible for PEP are referred for a baseline HIV test—if testing services are available—to confirm negative status before PEP initiation. The Malawi HIV programme implements a 3‐test algorithm for HTS for adults and children (Figure S1) [9]. All clients are counselled on voluntary and confidential testing, emphasizing their right to opt out without affecting access to other healthcare services. Informed consent is obtained from all HTS clients before testing. At each HTS session, regardless of PEP needs, a self‐reported history is documented, including the risk of exposure to HIV, HIV testing history (previous HIV test results and dates) and any prior antiretrivial (ARV) use, such as PEP, PrEP and ART. The risk of exposure to HIV is classified as high risk, ongoing risk, low risk or not assessed. “High risk of exposure to HIV” refers to a potential high‐risk HIV exposure event within the last 3 months (e.g. sexual assault, unprotected consensual sexual intercourse), while “ongoing risk of exposure to HIV” includes clients whose partners are HIV positive or on ART and HIV‐exposed infants (children aged < 2 years old born to HIV‐positive women). Clients who do not have high or ongoing risk of exposure to HIV are classified as having low risk. After HIV testing, clients receive post‐test counselling, which includes referral to ART, PEP or PrEP services.

PEP‐eligible individuals with an HIV‐negative result or unknown HIV status are initiated on PEP and receive a complete 30‐day PEP regimen, with a strong emphasis on adherence. The standard PEP regimen consists of weight‐based combinations: ABC/3TC + DTG for individuals weighing <30 kg (mainly children) and TDF/3TC/DTG for those weighing ≥30 kg [6]. An alternative regimen is an AZT/3TC‐based regimen used across all weight categories. Follow‐up visits are scheduled at 30 days post‐PEP initiation for adherence assessment and condom provision, and at 3 and 6 months for HIV testing to confirm the absence of HIV acquisition. In cases of sexual assault, emergency contraception is also provided.

### 2.3 Data collection for PEP and HTS

PEP initiations are documented in an improvised PEP register, but no dedicated monitoring and evaluation (M&E) tool captures follow‐up testing data. HTS data is captured in paper‐based HIV testing registers that are scannable with ScanForm, which are featured in the WHO strategic information guidelines to strengthen routine data for impact [10]. To enhance data quality, the ScanForm system has built‐in data validation checks that automatically identify and flag anomalies. The anomalies are disseminated through an online portal, with direct notifications sent to the relevant facilities for timely correction [11]. The resulting non‐identifiable client‐level electronic data is stored in a central data repository and integrated with DHIS2.

In accordance with Malawi MoH reporting guidelines, preliminary statistics are automatically generated weekly, and comprehensive monthly reports are shared with programme leadership by the fifth of each month. A dedicated dashboard provides HTS staff with real‐time access to performance metrics, including PEP data. The ScanForm technology improves HTS quality and M&E by generating reports highlighting data quality errors, missing information and violations of the 3‐test algorithm. As of July 2024, the ScanForm technology was rolled out in 74% (730/982) of the health facilities in Malawi.

### 2.4 Data sources

HTS data were extracted from the ScanForm HTS dataset, a central repository containing HTS records collected between November 2022 and July 2024 across Malawi. Each encounter in the dataset represented an individual instance of HIV testing, and some clients had multiple encounters during the study period.

### 2.5 Inclusion and exclusion criteria

We analysed two datasets. The first dataset, a cross‐sectional study component included all HTS encounters of individuals aged 2 years and older who reported PEP use at some point in their life. The second dataset, a cohort study, was designed to estimate HIV seroconversion. We constructed the HTS cohort using self‐reported PEP use history and HIV testing data, specifically the time since the last HIV test and previous test results. Since HIV testing data are recorded as individual HTS encounters rather than longitudinal client records, we implemented steps to establish a cohort of unique HTS clients (1) who returned for a follow‐up HIV test after recently completing PEP and (2) had no evidence of HIV acquisitions before PEP initiation and during PEP. This involved excluding the following HTS encounters:

1. Non‐negative previous HIV test results;
2. Baseline HIV test occurred more than 35 days before PEP initiation;
  (time between baseline test and PEP initiation was too long, more than a month)
3. Baseline HIV test occurred more than 7 days after PEP initiation;
  (suggests that the full PEP course was not completed)
4. Date of last PEP use was greater than 65 days ago;
  (new high‐risk events could have occurred since completing PEP)
5. Follow‐up HIV test was performed more than 95 days after PEP initiation.
  (new high‐risk events could have occurred since completing PEP)

### 2.6 Statistical data analysis

Descriptive statistics were used to report the characteristics of HTS clients included in the cross‐sectional study and cohort study components. Categorical variables were summarized using counts and proportions, while continuous variables were analysed using medians with interquartile range (IQR).

Using cross‐sectional data, we compared the characteristics and HIV test results between HTS clients who reported PEP use and those who did not. In the cohort analysis, we evaluated participant characteristics and HIV seroconversion. Follow‐up time was calculated as the interval between the previous baseline HIV‐negative test result and the follow‐up HIV test performed within 95 days after PEP initiation. Overall and gender‐specific HIV seroconversion rates were estimated using a Poisson regression model with an offset to account for varying follow‐up durations. HIV seroconversion rates, along with 95% confidence intervals (CI), were presented.

### 2.7 Ethical considerations

The study was approved by the National Health Sciences Committee in Malawi (protocol #: 23/12/4275). The committee waived the need for the client's informed consent because the study used routine programmatic data and did not include personal identifiers.

## 3 RESULTS

### 3.1 Characteristics and HIV test results of clients included in the cross‐sectional study component

Between November 2022 and July 2024, a total of 4,710,601 HTS encounters were recorded in HIV testing registers across 730 health facilities in Malawi and included in the cross‐sectional study component (Table 1). Of these, 21,298 (0.5%) encounters involved clients who reported PEP use. PEP users were more likely to be male, have tested HIV negative, be categorized as having “high HIV‐risk exposure” and have partners with unknown HIV status or HIV‐positive partners on ART (*p*<0.001).

**Table 1:** Characteristics and HIV results of clients for each encounter of HTS in Malawi by reported PEP use, November 2022–July 2024

	Total	Reported PEP use		Did not report PEP use		
	*N*	*n*	%	*n*	%	*p*‐values
**Total**	4,710,601	21,298		4,689,303		
Sex						<0.001
Male	1,479,979	13,595	63.8	1,466,384	31.3	
Female	3,228,919	7694	36.1	3,221,225	68.7	
Unknown	1703	9	<1	1694	<1	
Age at HTS (years)						
2−12	418,271	841	4.0	417,430	8.9	<0.001
13−19	819,027	1174	5.5	817,853	17.4	
20−29	2,006,062	10,933	51.3	1,995,129	42.5	
30−39	910,944	5888	27.7	905,056	19.3	
40−49	341,038	2020	9.5	339,018	7.2	
50−59	115,454	350	1.6	115,104	2.5	
60+	99,805	92	0.43	99,713	2.1	
Previous HIV test result						<0.001
Never tested	823,657	193	0.9	823,464	17.6	
HIV positive^a^	55,646	93	0.4	55,553	1.2	
HIV negative	3,827,589	20,972	98.5	3,806,617	81.2	
Invalid/inconclusive missing	3709	40	0.19	3669	0.1	
Median time (days) since the previous HIV test (IQR)	365 (152−730)	183	(61−365)	365	(152−730)	
Median time (days) since the last PEP (IQR)	−	213	(30−365)	−		
Classification of client's risk of exposure to HIV						<0.001
Low risk	3,193,525	7941	37.3	3,185,584	67.9	
Ongoing risk	786,150	6301	29.6	779,849	16.6	
High HIV‐risk exposure event in the last 3 months	318,654	6382	40.0	312,272	6.7	
Not assessed	412,272	674	3.1	411,598	8.8	
HTS access point						
Facility	4,316,205	19,715	92.6	4296.490	91.6	
Community	394,396	1583	7.4	392,813	8.4	
Partner's HIV status						
Negative	3,043,582	8854	41.6	3,034,728	64.7	
HIV status unknown	630,949	8156	38.3	622,793	13.3	
HIV positive, on ART	202,337	1319	6.2	201,018		
HIV positive, not on ART	7774	75	0.4	7699	0.2	
HIV positive, ART status unknown	3375	39	0.2	3336	01	
No partner	822,577	2855	13.4	819,722	64.7	
HIV test result given to the client						
HIV positive	91,190	426	2.0	90,764	1.9	
HIV negative	4,536,307	20,644	96.9	4,515,663	96.3	
Inconclusive/invalid	83,104	228	1.1	82,876	1.8	

Abbreviations: ART, antiretroviral therapy; IQR, interquartile range; HTS, HIV testing services.

^a^ Clients returned for confirmatory tests.

Among HTS encounters with PEP use, 13,595 (63.8%) were for males. Half of HTS encounters involving clients reporting PEP use were aged 20–29 years (*n* = 10,933, 51.3%), followed by those aged 30–39 years (*n* = 5888, 27.7%). The median time since the last PEP was 213 days (IQR 30–365). The risk of exposure to HIV among PEP users was: 7941 (37.3%) were classified as having a low risk of HIV exposure, 6301 (29.6%) had ongoing risk and 6382 (40%) had high risk. Regarding partners’ HIV status, 8854 (41.6%) of HTS encounters were for clients who reported having an HIV‐negative partner, 8156 (38.3%) for clients who did not know their partner's HIV status and 1319 (6.2%) for clients whose partners were HIV positive and on ART, 75 (0.4%) for clients with HIV‐positive partners who were not on ART and 39 (0.2%) for clients with HIV‐positive partners with unknown ART status.

### 3.2 HIV‐positive tests

**Figure 1:**
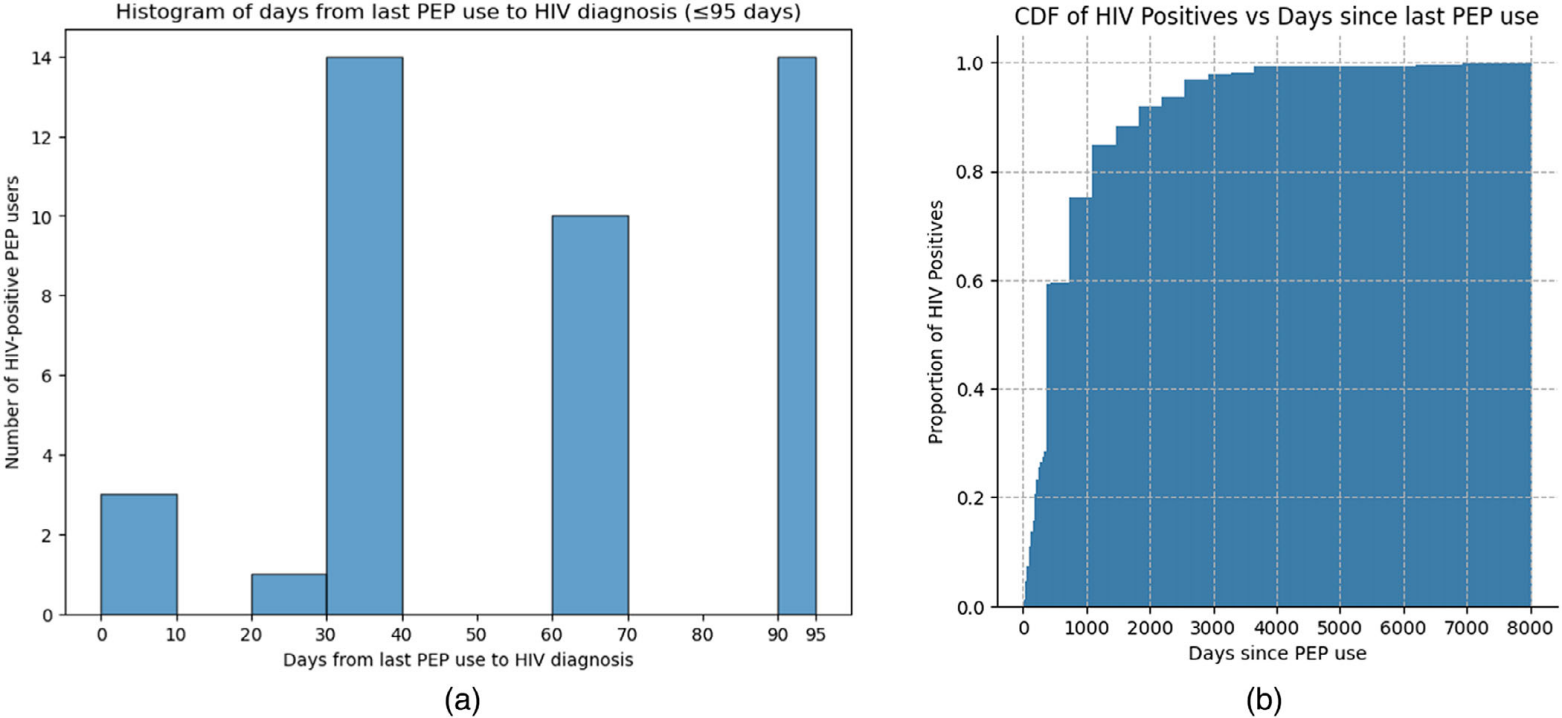
**(A) Histogram of days from last PEP use to HIV diagnosis (≤95 days) among HIV‐positive clients who reported PEP use. (B) Cumulative distribution function time between last PEP use and HIV diagnosis among all HIV‐positive clients who reported PEP use (*n* = 426) between November 2022 and July 2024**. Abbreviations: CDF, cumulative distribution function; PEP, post‐exposure prophylaxis.

The overall confirmed HIV positivity rate among clients who had ever used PEP was 2%, with 426 testing HIV positive. The HIV‐positive clients were clustered around 30, 60 and 90 days since the last PEP use, with very few positives reporting less than 30 days since the last PEP use (Figure 1A). Of the 426 HIV‐positive individuals, 10% were diagnosed within 95 days after the last PEP use (Figure 1B).

### 3.3 HTS cohort for PEP

**Figure 2:**
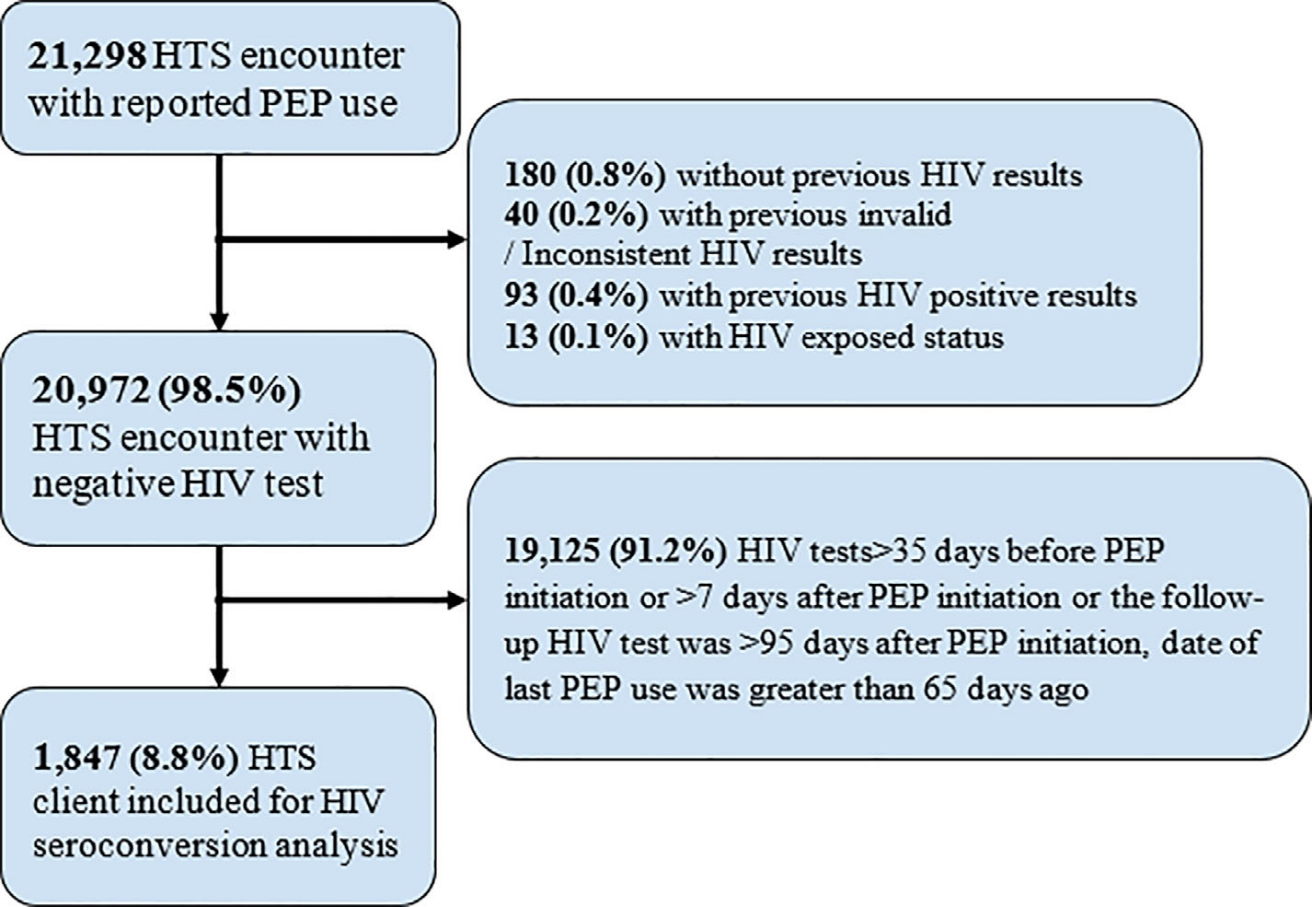
**Flowchart of clients who reported PEP use in Malawi between November 2022 and July 2024**. *Note*: HIV‐exposed status applies to children aged 2 years who had a previous rapid HIV‐positive test result. Abbreviations: HTS, HIV testing services; PEP, post‐exposure prophylaxis.

Of the 21,298 HTS encounters where clients reported PEP use, we excluded 326 (1.5%) encounters: 180 (0.8%) without a previous HIV status, 40 (0.2%) with invalid/inconsistent HIV status, 93 (0.4%) with previous HIV‐positive results and 13 (0.1%) were for children aged 2 years who had previous rapid HIV test (HIV‐exposed status) (Figure 2). Among the remaining 20,972 (98.5%) HTS encounters with negative HIV test results, 19,129 (91.2%) encounters were further excluded based on the following criteria: HIV tests were performed more than 35 days before PEP initiation or more than 7 days after PEP initiation or time since last PEP was more than 65 days, date of last PEP use was more than 65 days and follow‐up HIV testing occurred beyond 95 days post‐PEP initiation. A cohort of 1847 (8.8%) HTS clients met the inclusion criteria: baseline HIV‐negative results and a follow‐up HIV test within 95 days of PEP initiation.

### 3.4 Characteristics and HIV seroconversion among HTS clients included in the cohort study component

Among 1847 HTS clients included in the cohort analysis, 1055 (57.1%) were male (Table 2). Half (*n* = 927, 50.2%) was aged 20–29 years, followed by those aged 30–39 years (*n* = 460, 24.9%), while 292 (15.8%) were under 20 years. The median time since the previous HIV test was 30 days (IQR: 30–61), while the median time since the last PEP use was 3 days (IQR: 1–30). Nearly half of the clients (*n* = 892, 48.3%) were classified as having a low HIV risk. However, 581 (31.6%) reported ongoing HIV risk, 329 (17.8%) reported a high HIV‐risk event within the last 3 months and risk assessment was not conducted for 45 (2.4%). Regarding partner's HIV status, 752 (40.7%) HTS clients reported having an HIV‐negative partner, 625 (33.8%) indicated their partner's HIV status was unknown, 106 (5.8%) reported having an HIV‐positive partner and 364 (19.7%) reported having no partners.

**Table 2:** Characteristics of HTS cohort clients who reported PEP use, November 2022–August 2024

	*N*	%
Sex		
Male	1055	57.1
Female	792	42.9
Age at HTS (years)		
2−12	112	6.1
13−19	180	9.8
20−29	928	50.2
30−39	460	24.9
40−49	127	6.9
50+	40	2.2
Median time (days) since the previous HIV test (IQR)	30.0	(30−61)
Median time (days) since the last PEP (IQR)	3.0	(1−30)
Classification of client's risk of exposure to HIV		
Low risk	892	48.3
Ongoing risk	581	31.5
High HIV‐risk exposure event in the last 3 months	329	17.8
Risk assessment not done	45	2.4
Access point for HTS		
Facility	1789	96.9
Community	58	3.1
Partners HIV status		
Negative	752	40.7
HIV status unknown	625	33.8
HIV positive, on ART	100	5.4
HIV positive, not on ART	5	0.3
HIV positive, ART status unknown	1	0.1
No partner	364	19.7

Abbreviations: ART, antiretroviral therapy; HTS, HIV testing service; IQR, interquartile range; PEP, post‐exposure prophylaxis.

There was no association between sex and HIV exposure categories (Table 3). However, significant associations were found between age group and partner's HIV status with the client's classification of HIV exposure. Children aged 2–12 years were more frequently categorized as having a low risk of exposure to HIV (*n* = 78, 69.6%), while 12 (10.7%) had ongoing risk and 21 (18.8%) had a recent high HIV‐risk event in the last 3 months. The majority of individuals who reported having an HIV‐negative partner or no partner were frequently classified as having low risk (*n* = 566, 75.3% and *n* = 263, 72.3%, respectively). In contrast, individuals with partners whose HIV status was unknown or HIV positive were more often classified as having high or ongoing risk.

**Table 3:** HIV exposure category by characteristics of clients with baseline HIV test who reported PEP use, November 2022−July 2024

	Classification of risk of exposure to HIV					
	Total	High HIV‐risk exposure event last 3 months	Ongoing risk	Low risk	Risk assessment not done	Chi‐square
Sex						0.200
Male	1055 (57.1%)	192 (58.5%)	346 (59.6%)	488 (54.7%)	29 (64.4%)	
Female	792 (42.9%)	137 (41.6%)	235 (40.5%)	404 (45.3%)	16 (35.6%)	
Age group						<0.001
2–12	112 (6.1%)	21 (18.8%)	12 (10.7%)	78 (69.6%)	1 (0.9%)	
13–24	692 (37.5%)	130 (18.8%)	213 (30.8%)	333 (48.1%)	16(2.3%)	
25–49	1003(54.3)	174 (17.3%)	338(33.7%)	463 (46.2%)	28 (2.8%)	
50+	40 (2.2%)	4 (10%)	18 (45%)	18 (45%)	0 (0.0%)	
Partner's HIV status						<0.001
Unknown	625 (33.8%)	190 (30.4%)	372 (59.5%)	55 (30.4%)	8 (1.3%)	
Positive not on ART	5 (0.3%)	0 (0.0%)	3 (60.0%)	2 (0%)	0 (0%)	
Positive ART Unknown	1 (0.1%)	0 (0.0%)	1 (100%)	0 (0.0%)	0 (0.0%)	
Positive on ART	100 (5.4%)	12 (12.0%)	80 (80.0%)	6 (6.0%)	2 (2.0%)	
Negative	752 (40.7%)	69 (9.2%)	88 (11.7%)	566 (75.3%)	29 (3.9%)	
No partner	364 (19.7%)	58 (15.9%)	37 (10.2%)	263 (72.3%)	6 (1.6%)	

A total of five individuals seroconverted, yielding a seroconversion rate of 2.08 (0.87−4.99) per 100 person‐years: two males (1.42 [0.34−5.67] per 100 person‐years) and three females (3.02 [0.97−18.05] per 100 person‐years). Of the individuals who seroconverted, one reported a high‐risk event in the past 3 months, three had ongoing HIV exposure and one was categorized as low exposure. Regarding partner HIV status, two did not know their partner's HIV status, one had an HIV‐positive partner on ART, one had no partner and one had a partner who was HIV negative.

## 4 DISCUSSION

This study is the first globally to use a large‐scale national routine programme data to describe characteristics of HTS clients who reported PEP use and their HIV seroconversion rates. The use of nationally representative, real‐world data strengthens the relevance of the study and provides unique insights into national PEP implementation. Both the cross‐sectional and cohort components of this study showed that the majority of HTS clients who reported PEP use at any time prior were aged 20–29 years, with a higher proportion being male, and a sizeable proportion having substantial ongoing HIV exposure. In the cohort analysis, HTS clients with ongoing HIV exposure were those with unknown partner HIV status and those with HIV‐positive partners. The study found a high HIV seroconversion of 2% per year. The findings have several implications for the national PEP programme.

Similar to other studies [12, 13, 14, 15], the majority of the PEP users were young adults and males. In SSA, including Malawi, young adults are at elevated risk of HIV exposure due to increased sexual activity, often involving multiple or older partners [16]. The heightened risk awareness may drive more young adults to seek PEP following potential exposures such as condom slippage or breakage, sexual assault and unprotected consensual sexual intercourse. The gender disparity in PEP access may stem from structural and socio‐cultural barriers, including financial constraints, and stigma surrounding sexual violence disclosure despite women generally having higher health‐seeking behaviour [17, 18]. Women who experience sexual assault—especially from intimate partners—may not seek care [19]. Integrating PEP education and awareness campaigns with broader sexual and reproductive health services may empower young women to understand and access PEP.

Our study found that 4% of HTS clients who reported PEP use were children aged 2–12 years. Although this study did not collect specific details on HIV exposure, previous research highlights child sexual assault as a significant issue in Malawi [20, 21]. Additionally, anecdotal reports from facilities with one‐stop centres (those with a high number of child PEP users) reported that child sexual assault was the most common HIV exposure. In the past two decades, the Government of Malawi has collaborated with international organizations to combat violence against children, encourage reporting of sexual violence and establish national referral pathways connecting communities, law enforcement, social support networks and health facilities [22]. Given the significant number of children accessing PEP, scaling up educational campaigns on child sexual abuse prevention and strengthening national referral pathways to encourage prompt reporting are warranted.

The study identified a seroconversion rate of 2% per year, with five individuals seroconverting. While comparable to other studies [23, 24], the HIV seroconversion is relatively higher. Most studies on PEP, particularly among healthcare workers (occupational PEP), report low seroconversion rates, often less than 1% [25, 26]. In non‐occupational PEP use, seroconversion rates vary from 1% to 2.9% [27, 28], influenced by factors such as adherence to PEP regimens, ongoing HIV exposure and lack of proper follow‐up. Although PEP initiation timing and adherence were not determined, most individuals who seroconverted had ongoing HIV exposure (unknown partner HIV status or HIV‐positive partner), emphasizing the need for enhanced prevention measures, including condom use, partner HIV testing and transitioning from PEP to PrEP for those at continued risk [29, 30, 31].

Forty‐one percent of PEP users reported having HIV‐negative partners, with 75% classified as low risk, which contrasts with typical HIV risk profiles for individuals seeking PEP. There are two possible explanations for the discrepancy. First, underreporting of high‐HIV risk exposures, such as concurrent condomless partnerships or undisclosed sexual networks—may have resulted in misclassification of participants’ HIV risk status. People may underreport these events due to stigma and undesirable aspects [32, 33]. Second, PEP may have been initiated due to high HIV exposure events (e.g. occupational incidents or sexual assault), while subsequent risk assessment reflected the participants’ current low status rather than the prior high‐risk exposure that prompted PEP use.

Reporting PEP uptake, HIV exposure and completion rates in the Malawi HIV programme is challenging due to the lack of standardized tools. The HIV testing register data used in this study tracked whether clients had previously received PEP during HIV testing encounters, which was designed to monitor HTS client return rates. However, effective monitoring of PEP use remains an unmet need. Implementing a standardized PEP register that document HIV exposures, along with baseline and follow‐up testing results, would improve client tracking, enhance continuity of care, and ultimately help reduce seroconversion rates.

The study findings should be interpreted considering the following limitations. First, the prevalence of “ever used PEP” was calculated based on the HTS encounters, where some people may have received HTS multiple times, potentially leading to an overestimate. Second, reliance on self‐reported previous HIV testing history and use of PEP, which is subject to recall bias and misreporting, risks inaccurate estimation of ever‐used PEP. Third, the estimation of the HIV incidence may be influenced by the exclusion criteria, which may affect generalizability. Fourth, selection bias may affect the results, as HTS clients who returned for follow‐up HIV testing might differ from those who did not, potentially leading to an overestimation or underestimation of the true seroconversion rates. Lastly, some clients who were included in the HIV seroconversion analysis might have already acquired HIV but tested HIV negative before starting PEP, hence overestimating the observed seroconversion rates. Despite these limitations, the study accurately reflects current practices in PEP programme implementation and highlights critical areas for improvement.

## 5 CONCLUSIONS

Our findings show a high HIV seroconversion rate among PEP users. The majority of PEP users were male, suggesting a potential gap in reaching women. Younger individuals also represented a large proportion of PEP users. Many continued to engage in high HIV‐risk exposure behaviours, underscoring the need to promote condom use, partner HIV testing, PrEP access, alongside other evidence‐based safer sex strategies. PEP M & E tools are essential for effectively monitoring PEP uptake, adherence and seroconversion rates. Further research is necessary to understand the type of risk of exposure to HIV better.

## COMPETING INTERESTS

The authors declare that they have no competing interests.

## AUTHORS’ CONTRIBUTIONS

LG, WW, HT and TC designed the study. WW, ZZ, AD and DB conducted data analysis. HT, TC and AJ interpreted the data. HT, LG, WW and TC wrote a draft paper. All authors reviewed and approved the final paper.

## DISCLAIMER

The content is solely the responsibility of the authors and does not necessarily represent the official views of the Malawi Ministry of Health and the donors.

## Supporting information




**Figure S1**: 3‐test diagnostic algorithm for HIV in Malawi

## Data Availability

The dataset used in this study is available from the corresponding author upon reasonable request.
