# Supplementary figures and images for: Characteristics of individuals who received post‐exposure prophylaxis and HIV seroconversion in Malawi: an analysis of national routine HIV testing data
Source: J Int AIDS Soc. 2025 Jun 26;28(Suppl 1):e26473. doi: 10.1002/jia2.26473 (PMC12231657; doi:10.1002/jia2.26473)

**Supplemental Figure 1:** 3-test diagnostic algorithm for HIV in Malawi


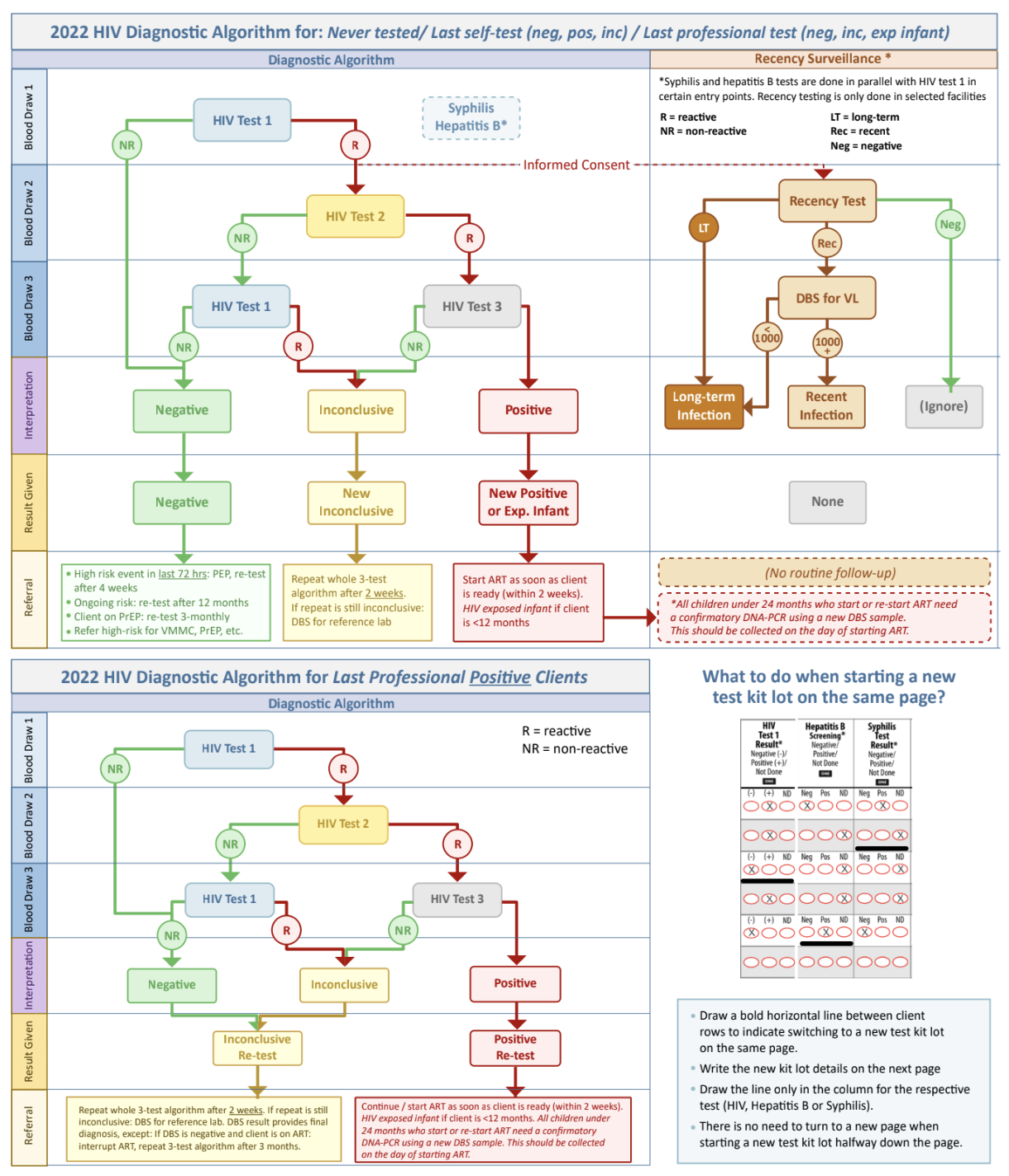

Supplement: Supplementary file 1 — Figure S1: 3‐test diagnostic algorithm for HIV in Malawi [file JIA2-28-e26473-s001.docx]
